# A qualitative analysis of female sport experiences in soccer

**DOI:** 10.3389/fspor.2025.1585654

**Published:** 2025-05-26

**Authors:** Kristy L. Smith, Noha El-Haj, Patricia L. Weir

**Affiliations:** Department of Kinesiology, University of Windsor, Windsor, ON, Canada

**Keywords:** relative age, sport participation, athlete engagement, sport dropout, birth advantage, sport sampling, community size

## Abstract

Relative Age Effects (RAEs) have the potential to be counterproductive to sport participation rates given the associated selection (dis)advantages and inequitable access to development opportunities for individuals of varying relative age. Previous work has predominantly been quantitative in nature and focused on male athletes, with only a few qualitative studies in the published literature. Thus, the purpose of this study was to examine relative age and sport engagement and dropout issues by conducting a qualitative analysis of post-adolescent, female athletes' experiences. An invitation to participate in a semi-structured interview (via phone) or online questionnaire (via Qualtrics) was distributed to a targeted sample of post-adolescent (18–19 years of age), current and past female soccer participants from Ontario, Canada (*N* = 15). Questions focused on reasons for participation and dropout, aspects of programs and relationships that facilitated or discouraged participation, player recommendations for encouraging future participation or reuptake of the activity, perception of abilities at various stages, involvement in other sports, location considerations, and age issues. The three stages of Côté's Developmental Model of Sport Participation were used to structure the questions in order to explore experiences occurring during specific stages of the athlete's developmental years. Hierarchical content analysis was used to identify raw data themes, which were grouped into higher order sub-themes and categories. Half year comparisons (H1 vs. H2) revealed similar themes reported by relatively older and younger participants, suggesting relative age was not the most important factor with respect to the players' experiences and decisions to continue in the sport when examined from a qualitative lens, although study design may have been a contributing factor. Engaged athletes reported a greater number of themes related to specialization in sport, and dropout athletes reported more negative sport experiences. Sport sampling at young ages (<12 years of age) was associated with ongoing sport participation into the post-adolescent years, with engaged athletes reporting involvement in a greater number of additional sports (beyond soccer), vs. the dropouts. Community size/characteristics reportedly impacted sport experiences, although no clear trends were ascertained. General recommendations for sport practitioners and recommendations for future research are discussed.

## 1 Introduction

Organized sport participation during childhood and adolescence has been associated with a variety of desirable outcomes including higher levels of cardiorespiratory fitness (1, 2), enhanced psychological and social health (3), and overall well-being (2). The benefits appear to extend beyond those attributable to physical activity alone, some of which are reinforced by the social nature of sport competition (4, 5). Unfortunately, high rates of sport dropout have also been reported (e.g., 6–8) with lack of enjoyment, low perceived competence, an increase in family and intrapersonal pressure, and physical factors (maturation and injury) being cited as primary contributors (9). Young girls may be at increased risk of dropout from sport participation (5, 10), which is concerning given that females are at increased risk for insufficient levels of physical activity when examined globally (11).

Within the context of organized sport, children are often grouped by chronological age. These age divisions are intended to promote a developmentally suitable environment to practice sport by providing age-appropriate access to training/tasks and competition (12, 13). However, subtle differences in physical, psychological, and emotional development among children grouped in a same-age cohort can lead to selection advantages and playing opportunities for the relatively older individuals (14, 15), as determined by their birthdate position with respect to an arbitrarily imposed date used for age groupings. Relatively younger individuals may not have these same opportunities to develop (13, 16, 17) and consequently may be more likely to struggle with issues of competence and reduced self-esteem (18). Relative age effects (RAEs) describe these potential (dis)advantages experienced by members grouped within a same-age cohort (19), which ultimately pose a threat to ongoing sport participation, as individuals who are disadvantaged (typically, the relatively youngest) may withdraw from sport altogether (20, 21) resulting in a reduced pool of talent for advancement to higher levels of competition.

The RAE-related advantage conveyed to those who are relatively older is considered to be present when an over-representation of relatively older players is observed among sport participants. For example, a team roster containing more athletes born in the earlier months of January, February, March… compared to athletes born in October, November, December, in a system that uses December 31st as a cut-off to group participants. These disproportionate birth distributions have been observed across a variety of sport and cultural contexts in both male and female samples (for a review, see 14, 22). With respect to team invasion sports, the risk of RAEs is influenced by biological characteristics (particularly during the maturation period), the sport's popularity, coaches' perceptions, and level of competition (23). For instance, RAEs have been observed in ice hockey (e.g., 24), soccer (e.g., 25), and handball (e.g., 26), to name a few. Individual, but still physically demanding activities, may also be affected with RAE patterns documented in sports such as tennis (e.g., 27), skiing (e.g., 28), and sprinting (e.g., 29). In contrast, sports that are more skill-based in nature (vs. physical prowess) tend not to exhibit RAEs, including golf (30) and shooting (31). Relative age effects have also been observed in the collegiate system within the US and Canada [see (32) and (33), respectively], and participation at this level may also serve to motivate individuals at young ages to seek higher levels of training and competition in an attempt to obtain athletic scholarships.

In light of the potential benefits associated with organized sport participation, effective strategies are needed to encourage ongoing engagement for developing youth. Creating an environment where athletes can thrive may encourage them to stay in sport and increase their overall well-being (2). Relatively younger athletes may feel isolated from their peers due to perceived differences in skill level and expectations (or lack thereof) placed on them in comparison to their relatively older teammates (34, 35). These differences may lead to conflicts between relatively older and younger athletes, contributing further to the feelings of isolation (34, 36). Thus, relative age has the potential to be counterproductive to this objective and therefore, the continued study of the RAEs is necessary to gain a thorough understanding of the contributing factors and identify meaningful ways to reduce the adverse outcomes. Yet, existing work investigating RAEs has been predominantly quantitative in nature, which is limited for understanding the intricacies of this multi-faceted, complex phenomenon. There are many aspects that still need to be analyzed (23), and qualitative studies may provide new insights into RAEs and relative age-related dropout. Existing qualitative studies have primarily focused on the experiences of coaches with respect to talent selection (37, 38); and only a few athlete-focused studies are available for review. Furthermore, female sport contexts have also been habitually understudied with respect to RAEs^1^, thus representing an important group for continued investigation.

Edwards and O'Donoghue (34) investigated the experiences of relatively older and younger (female) international-level netball players (ages 24–52 years; *N* = 13). The findings suggested that relatively older and younger athletes shared many motivations and obstacles, such as enjoyment of social and competitive aspects of sport, injury risk, and issues related to commuting. However, several factors were experienced more commonly and to a greater degree by the relatively younger athletes, including feelings of isolation from their team/governing bodies, being less developed and/or coordinated than older teammates, and conflicts with others on their team. These factors align with those reported to contribute to sport dropout by Andronikos et al. (36). Although their research was not tied specifically to RAEs, it was found that competing with teammates and receiving poor support from coaches and sport organizations was a significant contributor to dropout; issues that theoretically may be experienced to a higher degree by those who are relatively youngest across a variety of sport contexts.

A case study by Hancock (40) examined the experiences of relatively older and younger athletes, parents of both groups, and coaches from a youth (male) ice hockey team. Athletes were 14 and 15 years of age, born in the same calendar year. Several differences in the acknowledgment and acceptance of RAEs were noted in each individual's career. The relatively younger athletes and their parents were generally more knowledgeable of RAEs prior to the study and acknowledged the impact on their athletes, while the relatively older athletes and their parents reportedly knew less about RAEs initially and were slow to accept the potential implications of being relatively older. Interestingly, regardless of their opinion on RAEs, all parties believed that they did not have an effect on their own (child's) career. Coaches were aware of RAEs and claimed they did not allow them to influence their decision-making, yet they also held opinions that were (unknowingly) biased towards relatively older athletes (40). Ultimately, more work is needed to examine the experiences of the athletes themselves, and this work should be expanded to female athletes due to variation in physical growth and maturational timing, and associated social expectations/pressures that might be experienced differently between the sexes during development.

Given the lack of qualitative research examining RAEs in general, and more specifically the experience of athletes with respect to relative age-related influence on sport participation, the objective of this study was to explore relative age and sport engagement and dropout issues by conducting a qualitative analysis of post-adolescent, female athletes' experiences. Côté's Developmental Model of Sport Participation (DMSP) has been widely used in the participation literature (41) and thus, the stages of this model were used to organize the questions for the participants in order to examine various timepoints during development. Briefly, these stages include sampling a variety of sports at a young age (less than 12 years of age), specializing in a smaller number of sports during the adolescent years (or continued sampling in some cases; between 12 and 15 years of age), and investment in post-adolescence (16+ years of age; 42). A cohort of female athletes was identified through a provincial level organization, in which RAEs were observed across the pre- to post-adolescent years in Canada's most popular youth sport [i.e., soccer; Canadian Heritage (43) report]. Comparisons of “engaged” and “dropout” players, and relatively older and younger athletes were planned to examine the impact of relative age on sport experiences.

## 2 Methods

### 2.1 Research paradigm

The current study was designed from a reality-oriented, post-positivism paradigm; that being, an objective reality exists but is only imperfectly achievable (44, 45). The RAE phenomenon is notably complex (15, 23) and while this domain of research often seeks to promote equitable development for all young athletes, it is acknowledged that a complete understanding of the factors involved in athlete success/expertise may not be attainable.

### 2.2 Study design and theory

Côté's (42) three stages contained within DMSP were used to organize the questions for the semi-structured interview/questionnaire in order to explore experiences occurring during specific periods of an athlete's career. Specifically, the wording and organization of questions referred the participants to experiences occurring before 12 years of age, between 12 and 15 years of age, and current experiences at the age of 18–19 years, in sequential order. Edwards and O'Donoghue (34) also used these stages to identify when different participation and attrition motives were experienced, and to conceal that the study was concerned with RAEs. Thus, this study expands on the work of Edwards and O'Donoghue (34) with female, international-level netball players, by distinguishing between dropout and engaged players at various levels of competition in the sport of soccer.

The content of questions included reasons for participation and dropout, aspects of programs and relationships (i.e., with coaches, parents, and/or peers) that facilitated or discouraged participation, player recommendations for encouraging future participation or reuptake of the activity, perception of abilities at various stages, involvement in other sports, location considerations (e.g., community size), and age issues. These themes align with factors that have been proposed to affect youth sport experiences in previous research (34, 36, 46–48), and also allowed exploration of issues related to relative age. Participants were asked for their month of birth at the end of the interview/questionnaire, so as not to bias responses towards relative age, but collecting this information allowed an analysis of relatively older and younger athletes as outlined in Section 2.5. The DMSP stages align nicely with what is known about the impact of relative age at each developmental stage (22). The interview guide/questionnaire was adapted from Edwards and O'Donoghue (34; please refer to Supplementary Appendix A).

### 2.3 Participants and recruitment

Recruitment began during an earlier phase of a longitudinal, multi-study project, which examined the role of relative age, community size/density, and positive youth development (PYD) on female youth soccer participation. Briefly, a 1-year cohort (i.e., same birth year) was identified by Ontario Soccer and followed from age 10–16 years. Individuals who maintained participation into the final 2 years under examination (i.e., until a minimum age of 15 years) were recruited directly by Ontario Soccer to participate in an online survey examining developmental assets (further details can be found in (21); and (49). Following the completion of the online survey, participants were subsequently invited to provide their contact information if interested in participating in a separate future study. Fifty-three individuals initially indicated they were interested in participation, and the target age range of these individuals at the time of data collection was 18–19 years of age. A subsequent/direct invitation to participate in semi-structured, interview (via phone) or online questionnaire (via Qualtrics) was extended to this targeted subsample of current/former post-adolescent, female soccer players.

### 2.4 Data collection

Participants, which included current and former female soccer players, were given the choice to complete their interview on the phone (at a mutually agreeable time with the first author) or through an online questionnaire using the Qualtrics platform, which provided enhanced anonymity and allowed greater time for reflection. These procedures were cleared by the University of Windsor Research Ethics Board (REB #18-184). One participant opted for the telephone interview and fourteen selected the online format, for an overall sample of fifteen (*N* = 15). This sample size aligns with previous research, which has ranged from four to 25 participants (48, 50). Individuals who selected the online questionnaire were asked if they could be contacted for follow-up questions via email (13 participants agreed, one declined), whereas follow-up questions were asked during the interview session for the participant selecting the phone option. The interview completed via the phone was 14.11 min in length and the average length of time to complete the questionnaire on Qualtrics was 10.77 min (overall average 10.99 min), and varied depending on the amount of information that each participant chose to share.

### 2.5 Data analysis

The procedural steps of hierarchical content analysis were followed, as outlined by Sparkes and Smith (51). The basic unit of analysis was the individual participant. However, relative age (half year comparisons) and current status (dropout vs. engaged) were taken into consideration when evaluating and comparing emergent themes between groups. A preliminary review of the data was conducted by the authors to achieve familiarity (i.e., immersion). Two authors (K.S. and N.E.) manually identified raw data themes (i.e., meaning units from the transcribed text that contain one distinct piece of information) and labelled these themes with tags (52). The tags were manually grouped together into higher order sub-themes and categories, which were modified reflexively to accommodate new insights when required to find the best fit for the data (51). Each unique tag (i.e., raw data theme) was recorded a maximum of one time per participant. Themes, sub-themes, and categories were then organized into a table based on their hierarchical nature, while ensuring heterogeneity between each category. The number of occurrences for each theme were then tallied for the group of relatively older participants (H1; born in January through June based on the Dec. 31st cutoff used to organize youth soccer in Ontario, Canada; *n* = 8) and relatively youngest (H2; born in July through December; *n* = 7) to allow comparison. Additional details about the participants can be found in Supplementary Appendix B.

### 2.6 Data quality and trustworthiness

Two of the three researchers (K.S., P.W.) were familiar with RAEs within the context of sport. At the time of data collection, K.S. (Ph.D. Candidate) and P.W. (Faculty Supervisor) had conducted multiple studies on RAEs, including several involving female samples. Thus, they were able to identify valid themes in the raw data. However, the potential for bias associated with this familiarity was also recognized. Thus, two authors coded the data: one familiar with relative age research and qualitative analysis (K.S.); and one who had a general understanding of RAEs and sport participation research following the completion of undergraduate studies, but who was also new to this area of study and able to offer a fresh perspective on the data (N.E.). One case was coded by K.S. and N.E. in collaboration to provide training on the identification of meaning units within the data. This was followed by a second case coded independently and then discussed to provide additional feedback. The remainder of the cases were then fully coded independently by each author before further discussion took place. Agreement rates were 96.77% and 94.17% for the engaged and dropout participants, respectively. Disagreements were resolved through discussion and the coding structure was revised accordingly. A final review of the data was completed by the third author (P.W.), who was also familiar with relative age research.

### 2.7 Interpretation of the data

Analysis and interpretation sought principles for successful athletic development that could inform similar populations of post-adolescent female athletes. Developmental age (according to the stages of DMSP; 42), competition level (competitive vs. recreational), and community size^2^ (small/rural, mid-size, or large city) during participation were also taken into consideration. An exhaustive search of the literature was conducted following the final compilation of themes from the present study in order to compare it to any existing research that may be related. Findings were also compared to existing RAE-related hypotheses (e.g., maturation-selection hypothesis; 13, 14) and theoretical models (e.g., 35, 53).

## 3 Results

### 3.1 Overall findings

Raw data themes were compiled separately for “engaged” and “dropout” participants (i.e., participant status reported at the time of data collection, ∼18–19 years of age), and further delineated based on whether the participant was considered to be relatively older (H1) or relatively younger (H2) within this cohort. The hierarchical content analysis suggested 11 main categories in the data collected from engaged participants (comprised of 155 distinct meaning units) and 10 main categories for those classified as dropouts (representing 103 distinct meaning units). The themes, higher order sub themes, and categories are summarized in Tables 1, 2 for engaged and dropout participants, respectively.

**Table 1:** Athletes who were engaged at age 18–19 years (i.e., at time of data collection).

Theme^a^	H1 (*n* = 5)	H2 (*n* = 3)	Higher order sub theme	Category
Parents enrolled	3	3	Family influence	Initiation Determinants
Sibling(s) participated	2			
Family valued sport sampling		1		
Delayed engagement	1		Self/personal choice	
Friend played	1		Social network	
Invited to participate	1			
Low/negative perception of soccer specific skills	2	1	Soccer specific	Skill Perception vs. Peers (sampling years, age <12 years)
Average/neutral perception of soccer specific skills	1	2		
High/positive perception of soccer specific skills	1			
Not concerned with skill level	1			
(Hindsight) highly skilled	1			
Increasing confidence/skill level	3	1	Soccer specific	Skill Perception vs. Peers (specialization years, age 12–15 years)
Average skill	1			
Improved due to practice	1			
Comparison to other (talented) athletes		1		
Played with heart		1		
Increased level of competition	2	1	Specialization	Sport Progression (transition from sampling to specialization)
Increased training/practice	2			
Increased travel	1			
Year-round participation	1			
Increased investment	1	1		
Leadership role		1	Other	
Attrition of other players/reduced commitment of others	1			
Enjoyment	2	2	Enjoyment	Participation/Engagement Motives (at younger ages/past)
Fun	2	2		
Loved the sport/game	2	2		
Loved to play		2		
Observation of (older) others’ enjoyment	1			
(Enjoy) being active/running	2		Physical fitness motives	
Exercise/fitness		1		
Friends/relationships	4	3	Social motives	
Playing with a team/teammates/teamwork	2	1		
Family		1	Family motives	
(Older) sibling(s)	1			
Parent support/parents provided motivation	2	1		
Skill improvement/desire to improve	3	3	Personal improvement motives	
Goal setting		1		
Challenge		1		
Self-exploration		1		
Experience of success (e.g., score goal)		1	Success motives	
Excelled/skilled		1		
Competitive/competition	1			
Future aspirations (i.e., varsity team membership)	1			
Consistent coach	1		Coach related motives	
Coach support	1			
(Pressure due to) influence of/relationship with parents of teammates		1	Sport related issues	Potential Barriers to Participation
(Negative) sport politics		1		
Other sports (more enjoyable/fun)	1			
Dropout from other sports, due to…			In other sports	
- Injury	1			
- Travel issues	1			
- Time commitment	1			
- Loss of interest	1			
- Investment in soccer		2		
Skilled/confident	1	1	Self-awareness of skill	Current Skill Perception (at time of data collection 18–19 years of age)
Know (self) potential	1			
Room to improve		1		
Maintained skill, but not as refined (due to reduced play)	1	1		
Reached peak/stagnant (due to reduced practice/play)	1			
Average (due to injury)	1			
Reduced skill level (because aged out)	1			
Reduced importance of skill (replaced by fitness/fun)	1	1	Reduced emphasis on skill	
Enjoyment	1	1	Motives	Ongoing/Future Participation
Fun		1		
Love of the sport	4			
Love running	1			
Love competition	1			
Friends/relationships	3	2		
Being active/fitness	2	1		
Part of self-image/identity		1		
Transition to a less competitive context (e.g., Women's league, intramurals)	1	1	Intentions	
Only sport (thus, will continue)	1			
Opportunities available (current league, varsity team)		1		
Team constraints			Time constraints	Potential Barriers to Future Participation
- Work	1			
- School	1			
- Travel	1			
Surgery	1		Miscellaneous	
Academic goals (as a back-up)		1		
Uncertainty about future opportunities		1		
School success/pride		2	Experiences of success	Memorable Experiences
Winning games	2			
Scoring game winner/scoring goals	1	1		
Winning tournament	1	1		
Recognition/accolades (e.g., MVP)	1			
Team captain/leadership role		1	Leadership roles	
Co-captain with friend		1		
Disagreement with coach	1		Negative experiences	
Community support (lots of parents)	1		Network	Impact of Developmental Community (size, characteristics, etc.)
Friends as opponents		1		
Small town community/familiarity		1		
Always fun (with reference to small town)		1		
Lack of facilities	1		Resources	
Proximity to facilities		1		
(Positive) access to resources/environment		1		
Dispersion of talent (community size could not support # of clubs)	1			
(Negative) impact on recruitment (for varsity level	1			

^a^ Each theme captured a maximum of one time per participant.

**Table 2:** Athletes who had dropped out at age 18–19 years (i.e., at time of data collection).

Theme^a^	H1 (*n* = 3)	H2 (*n* = 4)	Higher Order Sub Theme	Category
Parent enrolled	2	2	Family influence	Initiation Determinants
Parent (as coach)		1		
Sibling(s) participated	1			
Interested in sport	1		Self/personal choice	
Knew others who played		1	Social network	
Program availability		2	Programming	
Low/negative perception of soccer specific skills	1	1	Soccer specific	Skill Perception vs. Peers (sampling years, age <12 years)
Average/neutral perception of soccer specific skills		1		
High/positive perception of soccer specific skills	1	1		
High/positive perception of general motor abilities (e.g., fast runner)	1	1	General abilities	
Superior skill level/lack of challenge	2	1	Soccer specific	Skill Perception vs. Peers (specialization years, age 12–15 years)
Improving/improved	1	2		
Average/appropriate skill level		3		
Declining skill level		1		
Rising to challenge (able to push themselves)		1		
Increased intensity/level of competition	1	3	Specialization	Sport Progression (transition from sampling to specialization)
Increased expectations from coaches	1		Coaching	
Familiarity (with other athletes)		1	Other	
Late start	1		Age issues	Age issues
Fun	2	1	Enjoyment	Participation/Engagement Motives (at younger ages/past)
Enjoyment	2	2		
Love of sport/soccer	1			
Love of competition	1	1		
(Enjoy) being physically active		1	Physical fitness motives	
Exercise/fitness	2	2		
Friends	2	3	Social motives	
Family influence	1		Family motives	
Playing with sibling(s)	1			
Quality time with parent (as coach)		1		
Opportunities to improve skill/skill improvement	3		Personal improvement motives	
Goal setting	1			
Superior abilities (provided motivation to focus)	1			
Lack of coach		1	Sport-related issues	Attrition (Dropout) Motives
Lack of teammates/participants		1		
Unfair playing time/coach decisions (at rec level)	1			
Lack of teams (for competition)	1			
Loss of engagement (boring)	1			
No team available (at older age)		1		
Feelings of not belonging to team	1			
Pressure to specialize in soccer (from coach)	1			
Off season training expectations	1			
Conflict with teammates	1			
Inappropriate coach	1			
Time constraints			Schedule conflicts	
- Work	1	1		
- School		1		
- Couldn't commit to travel	1			
Games too late	2			
Games too long	1			
Focus on different sport			Change of focus	
- Skill based reasons		1		
- Enjoyment		1		
(Choose to) focus on school		1		
Other interests		1		
Intramurals (at post-secondary institution)		1	Availability of opportunities	Future Participation (Intentions)
Recreational	1			
(For fun) with friends	1	1	Enjoyment	
In other sports (because more enjoyable)		1		
Tournaments			Tournaments	Memorable Experiences
- Winning		1		
- Fun		1		
- With friends	1			
Making friends/playing with friends	2	1	Social/team	
(Being part of) newly established team		1		
Practice		1		
Playing with sibling(s)	1		Family	
Network/friends who play	1	2	Network	Impact of Developmental Community (size, characteristics, etc.)
Play at school (recess)		1		
Status within the community	1			
Quality of team		1	Team	
(Explicitly stated) no impact of community	1		Other	

^a^ Each theme captured a maximum of one time per participant.

### 3.2 Participation motives—initial motivation

Overall, there were more similarities than differences between engaged and dropout participants regarding their experiences and motives for participation. At younger ages, participation/engagement motives were the most commonly identified category. For the engaged participants these represented 48 of the identified themes. The top three higher order sub themes included enjoyment (e.g., fun, love of the sport/game), social (e.g., friends/relationships, playing with teammates), and personal improvement motives (e.g., goal setting, challenge). Similarly, for those identified as dropout, the top higher order sub themes were also similar in nature and represented 28 of the identified themes, with enjoyment being the most highly cited, and social, physical fitness, and personal improvement motives noted equally. Family was also noted as a motivator for sport participation.

[EP14]:“My parents started me, but then I motivated myself. I liked being good at something and thought soccer could be the thing that was mine. The thing I enjoyed and I excelled at.”

[DP5]: “I liked playing and I got to hang out with my friends while I was there.”

When these motives were examined based on half-year, the raw data themes were well-balanced between H1 and H2, with no distinct differences observed in any aspect of this category for both engaged and dropout participants.

### 3.3 Participation motives—future motivation

While initial participation motives were similarly focused, motives for future participation revealed differences between engaged and dropout. For engaged participants, the second most frequently identified category was ongoing/future participation with the higher order sub themes of motives and intentions capturing the data (number of occurrences = 22). Consistent with motives at younger ages, enjoyment and social aspects were commonly cited. Several participants indicated that they intended to continue because opportunities were available, with reference to several competitive levels [e.g., (competitive) varsity teams vs. (recreational) intramurals].

[EP9]:“How fun it is. It's much less competitive in an intramural setting, and playing with friends who also have the primary goal of just having a good time is refreshing.”

In contrast, only 5 themes emerged for future motives within the group of players that dropped out. These include continuing to play recreational/intramural soccer, continuing in a different sport and the social aspects.

[DP5]:“No, I enjoy playing hockey more.”

What set the two groups apart was that those who did not continue in soccer identified attrition motives with a total of 22 distinct meaning units and higher order sub themes comprised of sport-related issues (e.g., lack of a coach, unfair playing time), schedule conflicts (e.g., could not commit to travel schedule, games too late/too long), and change of focus [e.g., (switch to) alternate sport, school, other interests]. With respect to negative sport-related issues, they were experienced to a greater degree by the dropout participants (vs. engaged counterparts) as expected, but there were a variety of themes captured as opposed to common/reoccurring issues experienced by multiple participants. Notably, Participant #12 expressed significant adversity with respect to a coach's expectations and pressure to specialize/train for soccer…

[DP12:]“I had some crazy coaches who took it too seriously. It got to a point where I no longer enjoyed it due to the screaming coach, rude girls, and ridiculous winter training expectations. Coaches should NOT expect children to focus on one sport in their teen years, especially because I didn't struggle on the team, I was one of the best. I was sad quitting but my coach gave me no choice. I cannot stress enough the importance to allow teenagers to explore other sports as training tends to be universal. Please tell coaches children should be allowed to play other sports regardless of what they are missing in the offseason as a result of it. In fact, they should encourage players to try other sports, or else too much of one sport just ruins the fun, as it did for me.”

Barriers to continued participation for the engaged participants included negative relationships, sport politics and issues related to other sports. For instance, Participant #9 stated…

[EP9:] “Becoming competitive in soccer did not leave any time for sports outside of soccer.”

In terms of future motivations and attrition motives/barriers to participation, two distinct differences emerged between H1 and H2 participants. For the engaged participants, four of five H1 participants mentioned “loving the sport” as compared to zero (of three) H2 participants. For the participants who dropped out, attrition motives related to sport (e.g., pressure, off-season expectations, coaching) and schedule conflicts were identified more frequently by the older players, while a change of focus (e.g., switch to new sport) were exclusively identified by the relatively younger H2 players.

### 3.4 Comparison of relatively oldest vs. youngest with respect to DMSP stages

Overall, there were more similarities than differences when comparing relatively older (H1) vs. younger (H2). There were no clear trends with respect to perceived skill level during the sampling years (i.e., less than 12 years of age); but the relatively older (engaged) members of this sample may have been more inclined to perceive themselves as skilled and reported opportunities for specialization between ages 12–15 years. Supporting this, within the category of Sport Progression, themes for engaged H1 participants included increased practice, competition, travel, and year-round training, which is commonly associated with early specialization in sport. For instance, Participant #2 reported the following…

[EP2]:“…it [soccer] got more competitive, more practices, I found myself travelling more to play on higher level teams where as when I was a kid it was closer to home…”

However, caution is needed when interpreting this data due to the low number of engaged H1 participants reporting within this sample (i.e., three of five H1 participants). These themes were not reported to the same degree among the dropout participants.

Interestingly, engaged participants were found to be greater samplers of sport at young ages when compared to the cohort of dropouts. Briefly, the DMSP (41, 42) recommends that children younger than age 12 years participate in a variety of activities before selecting their preferred sport(s) in later years. Engaged participants reportedly participated in an average of 3.25 other sports during the sampling years (range 0–6; two participants reported 6 other sports and both were H2). Further, engaged participants reported involvement in an average of 1.5 sports other than soccer during the specialization years (12–15 years; range 0–3 additional sports). By comparison, athletes who were reported dropouts at time of data collection participated in an average of 1 other sport (range 0–2) during the sampling years (<12 years), and an average of 1.14 sports other than soccer (range 0–2) during the specialization years (12–15 years). This suggests engaged participants sampled three times as many sports at age <12 years vs. the dropouts based on the average. Engaged participants were also more likely to report playing at a competitive level vs. dropouts.

### 3.5 Community size findings

With respect to community size, participants were primarily from a medium-sized community (overall, *n* = 11) as opposed to small (*n* = 3) or large (*n* = 1) communities. Themes related to social network (e.g., community support) and aspects of the built environment (e.g., proximity to facilities) were noted by both the relatively older and younger, with no clear trends. One participant discussed the negative impact of living in a (smaller) medium-sized community with respect to two issues: 1. Dispersion of talent and 2. Varsity-level recruitment, as evidenced in the following quotes from Participant #15:

[EP15]:“…we had two separate clubs…which I don't think our city was big enough to have, so that meant that you were splitting up the best players to each club depending on where you lived in the city and I think that that kind of hindered our success. If we had one club, we would have different levels of teams in the same age group and one super team of all the best players. We could pick and choose which kid would play at which level, but because we had two clubs, we split up where we were going.”

Her club team was not competitive against larger clubs and consequently, she would have preferred a combined, more competitive group of players to select from.

[EP15]:“…with the smaller/medium-sized city thing…I had wanted to play…I was interested in playing at the university level but with the smaller clubs, we didn't have access to the university coaches but if I had been playing at a larger club – it would have been easier to get in contact with the university coach…like I feel the university coaches are more invested in finding players at the larger clubs and it wasn't something that our club was ever like…hey, if you guys want to play at the university level, we’ll tell you how to get in touch with coaches, we’ll tell you the steps you need to be doing in order to have that opportunity.”

With respect to recruitment, the participant felt disadvantaged with respect to opportunities for participation at the post-secondary level and responsible for promoting herself as an athlete.

## 4 Discussion

### 4.1 Overall of findings related to participation

The purpose of this hierarchical content analysis was to examine relative age and factors contributing to sport engagement/dropout in a post-adolescent, subsample of female soccer players from Ontario, Canada. Additional determinants of youth sport participation (e.g., community size) were also explored, with consideration of the developmental stages outlined in the DMSP (42). Eleven categories emerged from the data provided by engaged participants (*n* = 8), and ten categories for dropouts (*n* = 7). The categories for each of the two groups were similar, although a few distinct differences were noted in the raw data themes. For instance, both engaged and dropout athletes reported similar influences related to initiation of the sport (e.g., parents, siblings), and similar variation with respect to skill perception vs. peers at young ages (less than 12 years).

Differences were noted within the transition from sampling to specialization (12–15 years) category, with engaged athletes reporting a higher number of themes related to specialization in sport (e.g., increased training/year-round training, increased travel). This may suggest that an increased level of commitment to sport during this period of development ultimately results in greater longevity/engagement as an athlete. This proposition is further supported by the reported participation trends of these athletes: that is, all engaged athletes were reportedly playing at a competitive level from ages 12–15 years; and 50% (four of eight) were still playing at a competitive level at the time of data collection which corresponds with 18–19 years of age (three reported participation at the recreational level, and the status of one participant was unknown).

Many participation motives were also shared between the two groups of engaged and dropout athletes, and the themes identified were consistent with previous research. For instance, enjoyment and support from parents, coaches, and/or peers have been found to be predictors of continued sport engagement (for a review, refer to 54). However, despite these similarities, the shared themes were not influential enough to keep the dropout group engaged. Perhaps for some, these motives to participate were overridden by negative experiences (discussed further in the following paragraph). Engaged and dropout participants also shared similar motives for ongoing and future participation, respectively, and similar themes related to their most memorable experiences in sport.

While both groups identified potential barriers to participation (for engaged) and attrition motives (for dropouts), differences were noted with respect to dropout players reporting a greater number of themes related to negative sport experiences (e.g., pressure to specialize, an inappropriate coach, conflicts with teammates). A lack of necessary sport-related resources was also noted [i.e., insufficient number of teammates/participants (to form a team), lack of a coach, no team available for older participants]. Andronikos and colleagues (36) reported similar contributions to sport withdrawal, including poor communication, inappropriate support, excessive pressure, and a win-focused environment. Likewise, Persson et al. (10) identified negative experiences, lack of suitable options within the sports club, high competitiveness/seriousness of training, and illness or injury as deterrents to continued participation. Additional factors contributing to dropout identified by Crane and Temple's review (9) include a lack of enjoyment, competing pressures, injuries, and perceived competence; these themes are consistent with the potential barriers and attrition motives reported by the participants in the current study.

### 4.2 Relative age-related differences (H1 vs. H2)

When the frequency of reported themes was examined based on relative age (H1 vs. H2) within the engaged and dropout groups, very few differences emerged. This may not be surprising given that participants were not explicitly asked about relative age-related experiences, nor were they informed that RAEs were under examination as part of study objectives. Furthermore, month of birth was intentionally requested at the end of the interview/questionnaire, so as not to bias the participant toward RAE-related themes during data collection. This procedure is consistent with the protocol used by Edwards and O'Donoghue (34). The engaged H1 participants did report a greater number of themes that may be connected to opportunities for early specialization (e.g., increased level of competition or travel, year-round training). Specifically, there were seven raw data themes that exemplified aspects of specialization reported by relatively older (H1) participants and only two instances provided by the relatively youngest (H2). These findings may be consistent with the maturation-selection hypothesis (e.g., 13, 14, 55) which suggests that those who are relatively older and consequently, further along in physical and psychological development, may be more likely to garner coaches' attention and be selected to higher level sport opportunities at earlier stages of development. However, it is difficult to ascertain the consistency of this trend in the current sample.

In general, the similarity of reported experiences mirror the findings of Edwards and O'Donoghue (34); however, they also found that relatively younger athletes experienced feelings of isolation from their team/governing bodies, being less developed and/or coordinated than older teammates, and conflicts with others on their team more often and to a greater degree than the relatively older athletes in their sample. These differences might be explained by the demographic characteristics of the two samples and/or context-related differences between the sports/regions examined in each study. Edwards and O'Donoghue (34) examined high-level (adult) netball players who had competed for one of two countries at some point during their careers; while the current sample included (post-adolescent) athletes from a variety of competitive levels across the province of Ontario, Canada.

The lack of relative age differences may suggest that RAEs are not an important determinant of athletes' experiences when examined from a qualitative perspective. Furthermore, relative age did not appear to be influential for identifying/predicting who would drop out in this sample; there was no evidence that RAEs impacted the players' experiences and decisions to continue in/disengage from the sport. However, it is also possible that RAEs are a less salient aspect of youth sport experiences, and these differences did not emerge as a result of the study design which aimed to explore youth sport experiences without biasing athletes towards relative age issues. A significant volume of literature has reported RAEs using quantitative methods across a wide variety of age groups, competitive levels, and sport contexts. But this quantitative work has not been able to answer the question why or fully unravel the underlying mechanisms contributing to observed trends that favour those with the earliest birthdates. Future qualitative work in this area will need to carefully consider how to explore RAEs as part of the lived experience of young athletes. Past relative age research has also been limited by the use of birth halves/quartiles and the associated loss of information with respect to participant outcomes (53); and the results of this study may be limited in the same manner.

### 4.3 The importance of sport sampling

The DMSP recommends that children participate in a variety of sports between the ages of 6–12 years, with an emphasis on motor development and fun (41, 42). This early diversification in sport has been suggested to foster fundamental skills for lifelong involvement and prolonged sport enjoyment (56, 57). This study supports the claim that sampling is important for ongoing engagement, with engaged participants reportedly sampling an average of three times more sports at age <12 years vs. the dropouts.

### 4.4 Community size

As outlined in the Results section, there were no clear trends between groups with respect to community size. However, ten of the fifteen participants contributing to this study acknowledged that community size/characteristics did impact their athlete experience in some manner (whether it be positive or negative). Further, a separate quantitative analysis of participation trends using the provincewide cohort associated with this subsample revealed participation differences based on community size and density categories (see 58). However, individual variation within community size and community density categories was evident upon detailed analysis. This area of study would greatly benefit from further qualitative work to determine how various community characteristics influence athlete experiences and associated decisions regarding participation.

### 4.5 General recommendations for sport

The findings of this study suggest that coaches have a significant role to play in athlete wellbeing. Reports of poor or inappropriate support, differing goals and expectations, and unfair/inappropriate behaviour from coaches were observed in the current data, and align with factors that have been found to contribute to decreased athlete enjoyment, engagement, and wellbeing with respect to sport (10, 34, 36). Coaches should employ developmentally appropriate strategies to provide athlete support and promote ongoing engagement (at any level) for the overall well-being of participants (59). With respect to RAEs, coach education has also been recommended and while additional work is needed to assess the empirical validity of specific interventions (60), it is likely important that coaches adjust their selection criteria to avoid relative age biases, and change the way athletes are categorized to promote equitable access to opportunities for all young athletes (23, 40).

### 4.6 Strengths, limitations, and future directions

This work contributes to an understanding of female sport experiences within the context of North American, developmental-level soccer. The sample size provided a detailed dataset for analysis within this particular sample of post-adolescent athletes; however, it was somewhat limiting when comparing subgroups (i.e., H1 vs. H2, community size, competition levels). Questions about past experiences may have been limited by memory biases, and responses in general may have been subject to limitations inherent in any type of self-report questionnaire, such as social desirability bias. Furthermore, verbal cues were available for the telephone interview, but not for the online Qualtrics questionnaire which may have negatively impacted the richness of the data that was collected from the participants who selected this method. This study also highlights the limitations of existing quantitative analyses when examining athlete experiences and RAEs; while it is certainly plausible that relative age differences impact sport participants in some manner during childhood and youth, the broader, multi-dimensional nature of sport participation needs to be considered when examining participation-related experiences (3, 53). Further, the perspective of participants who dropped out from soccer at younger ages (i.e., before age 15 years) were missing from this analysis and should be considered in future work to ascertain whether these athletes experience RAEs to a greater degree than the current sample.

### 4.7 Conclusions

This qualitative analysis has provided important insights with respect to the athletes' lived experiences and dropout/engagement behaviour within the sport of soccer. Relative age was not the most important factor with respect to the players’ experiences and decisions to continue in the sport when examined from a qualitative lens, with relatively older and younger participants reporting similar themes. Consistent with the tenets of the DMSP (42), sampling a variety of sports before 12 years of age appears to promote continued engagement into the post-adolescent years. Community size/characteristics impacted sport experiences, although no clear trends were ascertained.

## Data Availability

The datasets presented in this article are not readily available because access is not permitted due to privacy concerns. Requests and queries can be directed to smith43@uwindsor.ca.

## Footnotes

^1^The percentage of female studies (to male) has been estimated to vary between 2% (14) and 7.92% in team sports (39).

^2^Participants were asked to estimate the size of the community they currently lived in (1 = Rural/small town, e.g., less than 5,000 people; 2 = Medium-sized town or city, e.g., 5,000-500,000 people; 3 = Large city, e.g., More than 500,000 people; 4 = Not sure).
