# Supplementary material for: A qualitative analysis of female sport experiences in soccer
Source: Front Sports Act Living. 2025 May 26;7:1585654. doi: 10.3389/fspor.2025.1585654 (PMC12146340; doi:10.3389/fspor.2025.1585654)
Supplement: Supplementary file 1 [file Datasheet1.pdf]

Supplementary Appendix A – Interview/Questionnaire Guide

(Adapted from Edwards & O'Donoghue, 2014)

Sampling (< 12 years)

The participant will be instructed to think about her soccer participation when she was less than 12 years of age.

1. How did you get involved with soccer?
2. At what age did you start playing soccer?
3. What motivated you to play soccer at a young age
4. How did you perceive your ability at the time?

Probes

Can you elaborate/tell me more about…
- Age group issues
- The support provided by your family, friends, teammates, coach(es), and/or club?
- Participation and/or attrition motives?
- Community size, location, and/or travel issues

5. Did you play any other sports at this time?

Specializing / Continued Sampling (+12 years)

6. Was your experience playing soccer the same when you became a teenager?
   Probe: [If not] how did it change?
7. Were your motives the same as before?
8. How did you perceive your ability at this time?

Probes

Confirm competition level and age of selection processes (if applicable)

Can you elaborate/tell me more about…
- Age group issues
- The support provided by your family, friends, teammates, coach(es), and/or club?
- Participation and/or attrition motives
- Community size, location, and/or travel issues

9. Confirm any changes in the number of sports played, and reasons for any changes.

Investment or Continued Sampling (Current status at 18-19 years of age)

10. Are you currently playing soccer?
    Yes = *Engaged* participant; No = *Dropout* participant

Questions for Engaged Participants

11. At what level of soccer [competition] do you currently compete at?

12. What keeps you motivated to participate?
13. How do you perceive your ability at this time?
14. Do you plan to continue playing soccer?
    Probe: Why or why not?

Probes

Can you elaborate/tell me more about…
- Age group issues
- The support provided by your family, friends, teammates, coach(es), and/or club?
- Participation and/or attrition motives?
- Community size, location, and/or travel issues

Questions for Dropout Participants

12. What level of soccer [competition] did you participate in during your last year of play?
13. Why did you choose to stop playing?
14. What could have kept you involved in sport?
15. Do you plan to seek out opportunities to play in the future?
    Probe: Why or why not?

Probes

Can you elaborate/tell me more about…
- Age group issues
- The support provided by your family, friends, teammates, coach(es), and/or club?
- Participation and/or attrition motives?
- Community size, location, and/or travel issues

Closing Questions (For both Engaged and Dropout Participants)

15. As you reflect on previous years as a soccer player, does anything stick out as your most memorable soccer experience?
16. Do you currently live in a small/rural, medium-sized, or large/urban city?
    a. Have you always lived there?
    b. [In your opinion] did living in this community affect your experience as a soccer player in any way?
17. What is your current age?
18. What month were you born in?
19. Reaffirm consent to use the information provided by the participant.
20. Is there anything you wish to add or clarify?
21. Can I contact you in the future if I have any follow-up questions (If yes, confirm preferred method).

Supplementary Appendix B – Participant Demographics

Participant ID #	Month of birth [H1/H2]	Reported participation status at time of data collection (18-19 years of age)	Reported community size
1	July [H2]	Dropout	Large
2	April [H1]	Engaged	Medium
3	October [H2]	Dropout	Small
4	June [H1]	Engaged	Small
5	August [H2]	Dropout	Medium
6	May [H1]	Dropout	Medium
7	April [H1]	Dropout	Medium
8	April [H1]	Engaged	Medium
9	September [H2]	Engaged	Medium
10	November [H2]	Engaged	Small
11	February [H1]	Engaged	Medium
12	May [H1]	Dropout	Medium
13	October [H2]	Dropout	Medium
14	August [H2]	Engaged	Medium
15	March [H1]	Engaged	Medium
